# Supplementary material for: Clinical significance of pretreatment prognostic nutritional index and lymphocyte-to-monocyte ratio in patients with advanced p16-negative oropharyngeal cancer—a retrospective study
Source: PeerJ. 2020 Dec 1;8:e10465. doi: 10.7717/peerj.10465 (PMC7718802; doi:10.7717/peerj.10465)
Supplement: Supplemental Information 2 [file peerj-08-10465-s002.docx]

| Table S1. Association analysis between PNI/LMR and other clinical factors (n=142) | | | | | | | |
| --- | --- | --- | --- | --- | --- | --- | --- |
| Variable | | PNI | | *p* value | LMR | | *p* value |
|  |  | < 50.5 | ≧50.5 |  | < 4.45 | ≧4.45 |  |
| Age | <53 | 38 | 29 | 0.673 | 51 | 19 | 0.422 |
|  | ≧53 | 41 | 27 |  | 48 | 24 |  |
| ACE-27 | 0 | 55 | 35 | 0.537* | 66 | 27 | 0.898* |
|  | 1 | 21 | 17 |  | 28 | 14 |  |
|  | 2 | 3 | 4 |  | 5 | 2 |  |
| Smoking | no | 8 | 4 | 0.761* | 7 | 5 | 0.512* |
|  | yes | 71 | 52 |  | 92 | 38 |  |
| Betel nut chewing | no | 22 | 12 | 0.397 | 21 | 15 | 0.085 |
|  | yes | 57 | 44 |  | 78 | 28 |  |
| Alcohol drinking | no | 14 | 9 | 0.802 | 15 | 9 | 0.399 |
|  | yes | 65 | 47 |  | 84 | 34 |  |
| Clinical T classification | T1/2/3 | 29 | 18 | 0.583 | 35 | 17 | 0.635 |
|  | T4a/b | 50 | 38 |  | 64 | 26 |  |
| Clinical N classification | N0 | 13 | 9 | 0.953 | 17 | 8 | 0.837 |
|  | N1-N3b | 66 | 47 |  | 82 | 35 |  |
| Clinical TNM stage | III | 4 | 3 | .904* | 5 | 4 | 0.633 |
|  | IVA | 20 | 12 |  | 24 | 10 |  |
|  | IVB | 55 | 41 |  | 70 | 29 |  |
| Clinical ENE | negative | 44 | 28 | 0.513 | 56 | 21 | 0.396 |
|  | positive | 35 | 28 |  | 43 | 22 |  |
| Abbreviations: PNI, prognostic nutritional index; LMR, lymphocyte to monocyte ratio; ACE-27, Adult Comorbidity Evaluation-27; ENE, extranodal extension; *, fisher's exact test | | | | | | | |
